# Comparative Chemical Profiles of the Essential Oils from Different Varieties of *Psidium guajava* L.

**DOI:** 10.3390/molecules26010119

**Published:** 2020-12-29

**Authors:** Emad M. Hassan, Abd El-Nasser G. El Gendy, Ahmed M. Abd-ElGawad, Abdelsamed I. Elshamy, Mohamed A. Farag, Salman F. Alamery, Elsayed A. Omer

**Affiliations:** 1 Medicinal and Aromatic Plants Research Department, National Research Centre, 33 El Bohouth St., Dokki, Giza 12622, Egypt; emadnrc@yahoo.com (E.M.H.); sayedomer2001@yahoo.com (E.A.O.); 2 Plant Production Department, College of Food & Agriculture Sciences, King Saud University, P.O. Box 2460, Riyadh 11451, Saudi Arabia; 3 Department of Botany, Faculty of Science, Mansoura University, Mansoura 35516, Egypt; 4 Department of Natural Compounds Chemistry, National Research Centre, 33 El Bohouth St., Dokki, Giza 12622, Egypt; ai.el-shamy@nrc.sci.eg; 5 Pharmacognosy Department, College of Pharmacy, Cairo University, Kasr El Aini St., Cairo P.B. 11562, Egypt; mfarag73@yahoo.com; 6 Chemistry Department, School of Sciences & Engineering, The American University in Cairo, New Cairo 11835, Egypt; 7 Biochemistry Department, College of Science, King Saud University, Riyadh 11451, Saudi Arabia; salamery@ksu.edu.sa

**Keywords:** *Psidium guajava*, guava, volatile oils, chemometric analysis, chemical polymorphism, endogenous factors

## Abstract

Guava (*Psidium guajava*) leaves are commonly used in the treatment of diseases. They are considered a waste product resulting from guava cultivation. The leaves are very rich in essential oils (EOs) and volatiles. This work represents the detailed comparative chemical profiles of EOs derived from the leaves of six guava varieties cultivated in Egypt, including Red Malaysian (RM), El-Qanater (EQ), White Indian (WI), Early (E), El-Sabahya El-Gedida (ESEG), and Red Indian (RI), cultivated on the same farm in Egypt. The EOs from the leaves of guava varieties were extracted by hydro-distillation and analyzed with GC-MS. The EOs were categorized in a holistic manner using chemometric tools. The hydro-distillation of the samples yielded 0.11–0.48% of the EO (*v/w*). The GC-MS analysis of the extracted EOs showed the presence of 38 identified compounds from the six varieties. The sesquiterpene compounds were recorded as main compounds of E, EQ, ESEG, RI, and WI varieties, while the RM variety attained the highest content of monoterpenes (56.87%). The sesquiterpenes, *β*-caryophyllene (11.21–43.20%), and globulol (76.17–26.42%) were detected as the major compounds of all studied guava varieties, while *trans*-nerolidol (0.53–10.14) was reported as a plentiful compound in all of the varieties except for the RM variety. A high concentration of D-limonene was detected in the EOs of the RM (33.96%), WI (27.04%), and ESEG (9.10%) varieties. These major compounds were consistent with those reported for other genotypes from different countries. Overall, the EOs’ composition and the chemometric analysis revealed substantial variations among the studied varieties that might be ascribed to genetic variability, considering the stability of the cultivation and climate conditions. Therefore, this chemical polymorphism of the studied varieties supports that these varieties could be considered as genotypes of *P. guajava*. It is worth mentioning here that the EOs, derived from leaves considered to be agricultural waste, of the studied varieties showed that they are rich in biologically active compounds, particularly *β*-caryophyllene, *trans*-nerolidol, globulol, and D-limonene. These could be considered as added value for pharmacological and industrial applications. Further study is recommended to confirm the chemical variations of the studied varieties at a molecular level, as well as their possible medicinal and industrial uses.

## 1. Introduction

Since the beginning of humanity, plants have been used as the main resources of foods, medicines, clothing, and other goods [1]. Many pharmaceutical drugs are derived from plant resources with potent biological activities, along with the low side effects and costs [2]. There are more than 250,000 identified plant species worldwide; among them, 7000 species are cultivated plants that are used in various human activities [3] to provide a myriad of bioactive components, i.e., dietary fiber, minerals, vitamins, and diverse amounts of phytochemicals or secondary metabolites [4].

The guava tree (*Psidium guajava*; Family: Myrtaceae) is cultivated for its nutritive fruit characterized by high contents of minerals and vitamins [5]. However, other parts (the leaves, bark, and root) of the guava tree are used in traditional medicines to treat several diseases. The guava tree produces a large quantity of biomass that results from the continuous pruning process. This biomass—a waste or byproduct—can be considered as an added value where it can be integrated into the production of various bioactive compounds with pharmacological and industrial application [6]. Different extracts from the guava leaf exhibit potent biological activities, such as anti-inflammatory, antipyretic, neuroprotective, antihypertensive, hypolipidemic, anti-obesity, cardioprotective, antioxidant, hepatoprotective, antidiarrheal, anticancer, immune-strengthening, anti-osteo-renal, antimicrobial, antivirus, and antiplatelet aggregation activities [5,7,8,9,10,11]. In addition, several chemical investigations described the identification of several vitamins (A, C, B, E, and K), carbohydrates, tannins, triterpenoids, flavonoids, benzophenones, and phenolics [8,12,13,14].

The biological activities of *P. guajava* leaves usually correlate to its essential oils (EOs) and volatiles that represent the main constituents of the leaves. Many compounds can be characterized from the EOs that are extracted from guava leaves around the world, especially the terpenoids, such as limonene, *α*-pinene, eucalyptol, caryophyllene isomers, *α*-humulene, *γ*-murolene, selinene isomers, *β*-bisabolene, caryophyllene oxide, and epi-*β*-cubenol [5,6,7,15,16]. The EOs’ composition is reported to be affected by various exogenous factors, such as precipitation, light, season, altitude, and soil characteristics. In addition, various endogenous factors such as anatomical, physiological, and genetic characteristics can modify either the qualitative or quantitative amounts of the EOs’ chemical compounds [17,18,19]. Chemical polymorphism is a phenomenon wherein the same species show variation in the chemical composition of the bioactive compounds [20,21]. This phenomenon is well known in the EOs of various plants [20,22,23,24]. The study of the plants’ variations in chemotypes is essential from a taxonomic point of view, as well as for agronomic and pharmacological applications [6,25]. The chemical polymorphism of the EOs from 22 genotypes of *P. guajava* grown in two Brazilian environments was observed by de Souza et al. [6]. However, the chemical polymorphism in *P. guajava* that grows in Egypt is not well studied. Therefore, the present work aims to (i) construct the chemical profiles of EOs extracted from the leaves of six cultivated varieties of *P. guajava* growing under similar environmental conditions in Egypt, and (ii) to establish a chemical-based relationship among the six varieties using chemometric analysis.

## 2. Results and Discussion

### 2.1. Chemical Profiles of the EOs from Different Varieties of P. guajava

The EOs were extracted via hydro-distillation from the leaves of six varieties of guava: Red Malaysian (RM), El-Qanater (EQ), White Indian (WI), Early (E), El-Sabahya El-Gedida (ESEG), and Red Indian (RI). The extracted EOs showed considerable variation in the yields, wherein they produced 0.48, 0.25, 0.21, 0.19, 0.18, 0.15, and 0.11% (*v/w*) for ESEG, RI, E, RM, WI, EQ, and RT, respectively. The oil obtained from the ESEG guava variety was comparable to that extracted from varieties of *P. guajava* cultivated in Pakistan (0.60%) [15], Tunisia (0.66%) [26], Brazil (0.40%) [6], and Oman (0.38%) [16]. In contrast, other studied varieties attained lower yields compared to other investigated varieties (Brazilian, Tunisian, and Omani). These variations could be related to seasonal variations, climatic conditions, or habitat [27,28,29,30,31]. The high yield in the ESEG variety showed that it is a premium variety for the production of guava essential oil.

**Figure 1:**
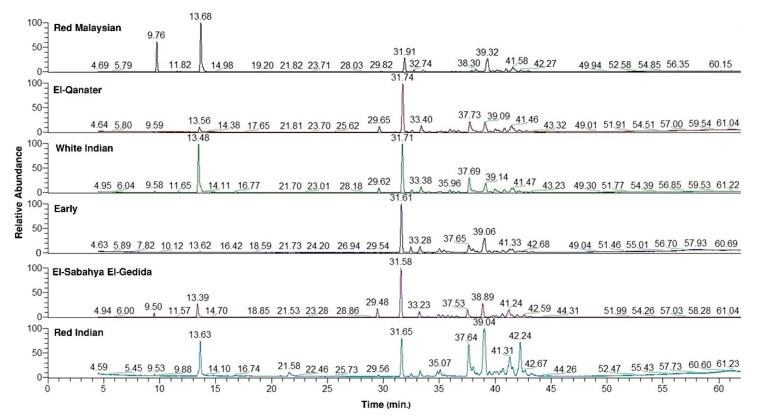
Gas chromatography–mass spectrometry (GC-MS) chromatogram of the essential oils (EOs) from the leaves of the six studied varieties of *Psidium guajava*.

**Figure 2:**
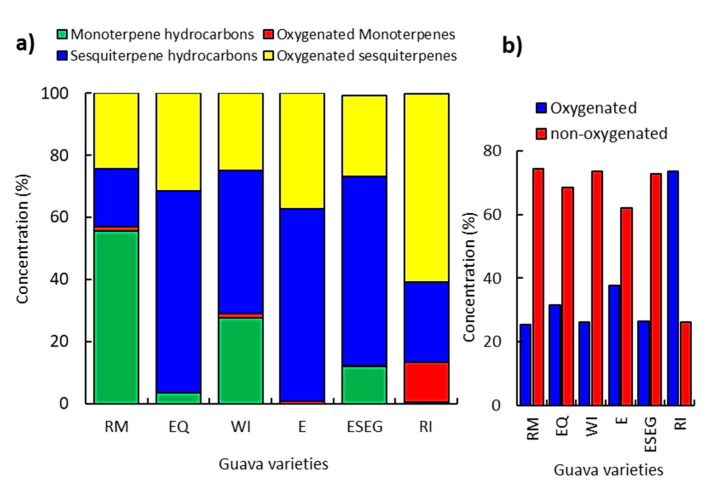
Relative percentile levels of the different classes of the identified compounds of the six studied varieties of *P. guajava*; (**a**) the four specified classes, and (**b**) the oxygenated and non-oxygenated components.

The GC-MS chromatograms revealed substantial variations among the six different varieties (Figure 1). The GC-MS analysis revealed that the chemical compounds can be categorized under four classes (Figure 2). The sesquiterpenes of the E variety were classified into sesquiterpene hydrocarbons (62.15%) and oxygenated sesquiterpenes (37.15%). Meanwhile, the ESEG variety attained 60.95% as sesquiterpene hydrocarbons and 26.11% as oxygenated sesquiterpenes (Figure 2a).

On the other side, the RM variety attained the highest content of monoterpenes (56.87%), composed mostly of monoterpene hydrocarbons (55.72%), while oxygenated monoterpenes were minor (1.15%). The RI, E, EQ, WI, and ESEG varieties attained 28.88, 13.41, 12.22, 3.55, and 0.68% of the monoterpene compounds (Figure 2a). In general, RI attained the highest content of oxygenated compounds (73.72%), followed by E (37.83%), EQ (31.49%), ESEG (26.38%), WI (26.24%), and RM (25.51%). In contrast, the RM, WI, and ESEG varieties exhibited 74.48, 73.72, and 72.90% as non-oxygenated compounds, which suggested that terpene hydrocarbon biosynthesis is more activated in these varieties (Figure 2b).

The observed variations among the different varieties could be ascribed to genetic variability [32]. Therefore, this chemical polymorphism of the studied varieties supports that these varieties could be considered as genotypes of *P. guajava*. This phenomenon is known to exist for other species such as *Thymus carnosus* [23], *Salvia fruticose* [20], *Calotropis procera* [17], *Origanum libanoticum* [24], *Origanum syriacum* [20], and *Cinnamomum osmophloeum* [22]. The exogenous factors such as precipitation, light, season, altitude, and soil characteristics can modify either the qualitative or quantitative amounts of the chemical compounds in the EOs [18,19,29,33]. However, the samples of the different varieties in the present study were collected from the same location in the same period; therefore, the exogenous factors can be excluded as controlling factors.

The chemical profiles of the EOs of different *P. guajava* varieties are shown in Table 1. A total of 38 chemical compounds were identified in the EOs of the studied guava varieties. The WI variety attained the highest number of compounds (29), while RM, ESEG, RI, E, and EQ had 28, 26, 25, 23, and 20 compounds, respectively. This composition was relatively higher than those reported for the Tunisian variety [26].

The sesquiterpenes *β*-caryophyllene and globulol were detected as major compounds of all studied guava varieties, while *trans*-nerolidol was reported as a major compound in all except for the RM variety (Table 1). The preponderance of caryophyllene in these varieties was in accordance with those reported by de Souza et al. [6], where they investigated 22 guava genotypes grown in two environments. However, globulol was not detected in the genotypes of de Souza et al.’s [6] study. Although Arain et al. [15] reported that *P. guajava* leaves collected from Pakistan present an excellent source of *β*-caryophyllene, our study revealed that the ESEG and E varieties of *P. guajava* had approximately twice the amount of *β*-caryophyllene compared to that of the Pakistani variety.

The EOs of the RM guava variety showed the presence of D-limonene, *α*-pinene, globulol, and *β*-caryophyllene, and they are represented by 33.96, 20.58, 14.13, and 11.21%, respectively. In the EQ variety, the main compounds detected were *β*-caryophyllene, globulol, *trans*-nerolidol, and *α*-copaene, recorded at 43.20, 10.57, 9.03, and 6.71%, respectively (Table 1). The EQ EO results were similar to the figures reported for the Pakistani variety [15]. According to these results, D-limonene and *α*-pinene might be assigned as a chemo-taxonomical fingerprint for the RM guava variety, while *trans*-nerolidol and *α*-copaene can be assigned for the EQ variety.

On the other side, *β*-caryophyllene (30.33%), D-limonene (27.04%), *trans*-nerolidol (8.27%), and globulol (6.17%) were reported as significant compounds in the WI variety. *β*-caryophyllene (43.12%), globulol (18.47%), and *trans*-nerolidol (5.81%) were the major constituents of the E variety of *P. guajava*.

The ESEG variety showed a high content of sesquiterpenes, exemplified by *β*-caryophyllene (38.42%), globulol (10.75%), *α*-copaene (7.00%), and *trans*-nerolidol (5.39%). Moreover, the RI variety was characterized by a preponderance of globulol (26.42%), *β*-caryophyllene (13.40%), eucalyptol (10.89%), eudesm-7(11)-en-4-ol (10.59%), and *trans*-nerolidol (10.14%). In our study, the six varieties were cultivated in the same garden with the same cultivation, soil, and climate conditions that directly affect the EO chemical compositions [18,19,29,33]. Thus, these observed variations in the chemical composition of the studied varieties confirmed the polymorphism phenomenon. Consequently, these varieties can be considered as different chemotypes of *P. guajava*. In addition, these variations could be attributed to endogenous factors such as individual genetic variability [32].

*β*-Caryophyllene is reported to possess anticancer, analgesic [34], anticonvulsant [35], anti-inflammatory [36], antioxidant, and antimicrobial activities [37], and its abundance, especially in the E, EQ, and ESEG varieties, makes them a possible source of this compounds. As a result, *β*-caryophyllene was described as a potential agent to treat several diseases due to its activity [38]. In addition, globulol, which was the highest compound in the RI variety, is reported to have various biological activities, such as antifungal [39] and antibacterial activities [40]. RI is also abundant in nerolidol and reported to possess antileishmanial [41], antiparasitic [42], antimalarial [43], and antimicrobial activities [44]. 

The biologically active monoterpenes *α*-pinene and limonene were found in the main compounds of the RM variety. *α*-pinene was described as the main component of most EOs derived from the plant kingdom [45,46]. This compound is integrated as a basic intermediate in bakery and chilled dairy products [47]. Several studies report that the isomers of pinenes, especially *α*-pinene, have various biological potentialities such as anti-inflammatory, antimicrobial, anticancer, antiviral, flavor, fragrance, antiallergy, and fungicidal activities [45]. On the other hand, D-limonene was reported as a safe anticancer agent, particularly for breast cancer [48]. As a result, the guava leaves’ high biomass yield could be considered a rich resource for these effective and sustainable bioactive compounds.

### 2.2. Multivariate Data Analysis of the EOs GC-MS Dataset

**Figure 3:**
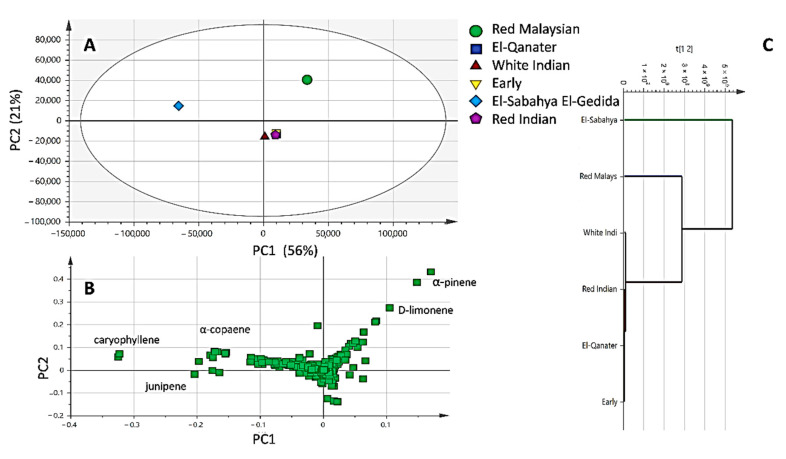
Principal component analysis (**A**) score plot, loading plot (**B**), and hierarchical clustering analysis (**C**). The model explains 77% of the total variance prescribed by principal components (PC1 and PC2).

Although differences in chromatographic patterns were observed among essential oil specimens, we attempted to categorize them in a holistic manner using chemometric tools. Principal component multivariate data analysis (PCA) was applied to model the EO compounds dataset (Figure 3A) and extracted using Metabolomics Ion-based Data Extraction Algorithm (MET-IDEA), and led to the detection of 867 Mass Spectral (MS) signals. The model accounted for 77% of the total variance described by principal components (PC1 and PC2). The PCA score plot (Figure 3A) revealed the distant separation of RM with positive score values along PC1 (right in PC1), whereas the ESEG variety was positioned on the other side with negative score values along with PC. On the other side, all other varieties were clustered together in the center of the PCA and had positive score values. Moreover, the examination of the loading plot revealed that *α*-pinene and limonene contributed the most to oil segregation and were more abundant in the RM variety. In contrast, the position of the ESEG variety was attributed to its abundance in sesquiterpenes, i.e., caryophyllene, *α*-copaene, and junipene. Hierarchical clustering analysis further confirmed the EOs segregation pattern, where RM and ESEG varieties were placed separately away in the dendrogram (Figure 3C).

## 3. Materials and Methods 

### 3.1. Plant Materials Collection and Preparation

The fresh, healthy, and well-developed (mature) leaves of the six guava (*P. guajava*) varieties were collected during the fruiting period (June 2019) from the same garden in Almansouria, Alharam, Egypt. These varieties were characterized as Red Malaysian (RM), El-Qanater (EQ), White Indian (WI), Early (E), El-Sabahya El-Gedida (ESEG), and Red Indian (RI). The garden is located in a semi-urban area, and the soil in the garden is loamy. The climate of the study area has an average temperature of 30–35 °C and average relative humidity of 60%. All of the collected varieties were authenticated by Mohamed El Gebaly, Professor of Taxonomy at the El-Orman Garden and National Research Center. The leaves were dried in the shade at room temperature (25 ± 3 °C) for two days before they were ground into a fine powder and packed in paper bags at −4 °C until further analysis [30]. Because air-drying aromatic plants at high temperature causes isoprenoid loss [49], leaf samples were dried in a shady place at room temperature, and all samples were treated with same procedures to avoid bias.

### 3.2. EOs, Extraction, GC-MS Analysis, and Components Characterization

The air-dried leaves (200 g) of the six varieties were subjected separately to hydro-distillation using Clevenger-type apparatuses (Shiva Scientific Glass Private Limited, New Delhi, India) for three hours. The oil layer was collected using hexane, dried with 0.5 g of sodium sulfate (anhydrous), and stored in glass vials until GC-MS analysis. The six extracted EOs were separately analyzed via the GC-MS technique using the GC-MS instrument (THERMO Scientific ™ Corporate, Waltham, MA, USA) at the Department of Medicinal and Aromatic Plants Research, National Research Center, Egypt [17]. The specifications of the used GC-MS instrument were adjusted according to the following conditions: TRACE GC Ultra Gas Chromatographs (THERMO Scientific™ Corporate, Waltham, MA, USA), lined with a Thermo Scientific ISQ™ EC single quadrupole mass spectrometer. The GC-MS system was equipped with a TR-5 MS column with dimensions of 30 m × 0.32 mm, i.d., 0.25 μm film thickness. Helium was used as carrier gas at a flow rate of 1.0 mL/min with a split ratio of 1:10 using the following temperature program: 60 °C for 1 min, rising at 4.0 °C/min to 240 °C, and held for 1 min. Both the injector and detector were held at 210 °C. An aliquot of 1 μL of diluted samples in hexane (1:10, *v/v*) was always injected. Mass spectra were recorded by electron ionization (EI) at 70 eV, using a spectral range of *m/z* 40–450.

Chemical constituents of the EOs under investigation were characterized by Automated Mass spectral Deconvolution and Identification (AMDIS) software, version 2.71 (Gaithersburg, MD, USA) (www.amdis.net), retention indices (relative to *n*-alkanes C_8_–C_22_), and comparison of the mass spectrum with authentic compounds (if available) from the Wiley spectral library collection and NIST library database (Gaithersburg, MD, USA; Wiley, Hoboken, NJ, USA).

### 3.3. GC-MS Multivariate Data Analyses

The chemical compounds of the identified EOs were extracted using MET-IDEA software with default parameter settings for GC-MS [50]. The aligned peak abundance data table was further exported to principal component analysis (PCA) using the SIMCA-P version 13.0 software package (Umetrics, Umeå, Sweden). All variables were mean-centered and scaled to the Pareto variance.

## 4. Conclusions

The GC-MS analysis of the six studied varieties of *P. guajava* revealed a substantial variation either in the quantity or quality of their EOs’ chemical composition. This variation reflects the chemical polymorphism phenomenon, and these varieties are considered to be different chemotypes. Based on the main compounds and the PCA analysis, it is evident that some main compounds such as *β*-caryophyllene and globulol were reported in all studied varieties, while *trans*-nerolidol was reported as a major compound in all varieties except for the RM variety. Other major compounds characterize specific varieties; for example, *α*-pinene and limonene characterize the RM variety, and caryophyllene, α-copaene, and junipene distinguish the ESEG variety. Therefore, these compounds could be used as a chemical fingerprint to identify these varieties. In practical terms, these major compounds are biologically effective compounds with various activities. The large biomass of guava trees that results from the pruning process, which is considered a waste or byproduct, can be a potential source for these important compounds. The characterization of chemotypes in cultivated plants is crucial for agricultural applications, chemistry purposes, and pharmacological uses. Further study is recommended to characterize the studied varieties at the molecular level to confirm their chemotaxonomic differences.

## Figures and Tables

**Table 1:** Chemical compositions of the essential oils (EOs) extracted from the leaves of six varieties of *P. guajava*.

No	Compound Name	RT	KI_Exp_	KI_Lit_	*P. guajava* Varieties						Identification
					RM	EQ	WI	E	ESEG	RI	
**Monoterpene hydrocarbons**											
1	*α*-Pinene	9.76	940	939	20.58 ± 0.60	---	0.48 ± 0.03 *	---	2.76 ± 0.04	0.48 ± 0.01	MS, KI
2	*β*-Pinene	11.57	982	980	0.49 ± 0.03	---	---	---	0.09 ± 0.02	---	MS, KI
3	*β*-Myrcene	11.82	990	991	0.54 ± 0.03	---	---	---	---	---	MS, KI
4	δ-3-Carene	12.98	1009	1010	0.15 ± 0.01	---	---	---	---	---	MS, KI
5	D-Limonene	13.68	1033	1031	33.96 ± 0.70	3.55 ± 0.07	27.04 ± 0.52	---	9.10 ± 0.08	---	MS, KI
**Oxygenated monoterpenes**											
6	Eucalyptol	13.91	1034	1033	0.97 ± 0.0	---	0.63 ± 0.03	0.68 ± 0.02	0.16 ± 0.01	10.89 ± 0.21	MS, KI
7	Linalool	16.75	1104	1104	---	---	0.26 ± 0.02	---	---	0.75 ± 0.02	MS, KI
8	*α*-Terpineol	21.86	1195	1197	0.18 ± 0.01	---	0.09 ± 0.01	---	0.11 ± 0.01	1.29 ± 0.04	MS, KI
9	Geraniol formate	22.65	1313	1312		---	0.38 ± 0.02	---	---	---	MS, KI
**Sesquiterpene hydrocarbons**											
10	*α*-Copaene	29.82	1376	1376	0.93 ± 0.03	6.71 ± 0.08	3.41 ± 0.06	0.50 ± 0.03	7.00 ± 0.08	0.49 ± 0.03	MS, KI
11	*α*-Gurjunene	30.90	1411	1409	---	---	---	---	0.13 ± 0.01	---	MS, KI
12	*β*-Caryophyllene	31.91	1419	1418	11.21 ± 0.33	43.20 ± 0.24	30.33 ± 0.31	43.12 ± 0.26	38.42 ± 0.34	13.40 ± 0.11	MS, KI
13	Aromadendrene	32.74	1437	1439	1.55 ± 0.06	1.48 ± 0.04	1.55 ± 0.04	4.78 ± 0.06	0.02 ± 0.01	1.21 ± 0.02	MS, KI
14	*cis*-Muurola-3,5-diene	32.97	1452	1450	---	---	0.41 ± 0.01	---	---	---	MS, KI
15	*α*-Humulene	33.55	1454	1455	1.27 ± 0.04	4.99 ± 0.07	3.56 ± 0.04	4.84 ± 0.07	4.03 ± 0.05	1.96 ± 0.06	MS, KI
16	*β*-Copaene	34.27	1460	1460	0.19 ± 0.01	0.49 ± 0.02	0.62 ± 0.02	0.58 ± 0.03	0.50 ± 0.03	---	MS, KI
17	*β*-Selinene	34.85	1486	1485	---	---	0.21 ± 0.01	0.41 ± 0.01	---	1.31 ± 0.02	MS, KI
18	*α*-Bisabolene	35.05	1503	1504	0.20 ± 0.02	---	---	3.27 ± 0.05	2.08 ± 0.04	2.02 ± 0.05	MS, KI
19	*β*-Bisabolene	35.34	1510	1509	0.20 ± 0.02	0.85 ± 0.02	0.72 ± 0.02	1.99 ± 0.04	1.55 ± 0.06	0.59 ± 0.03	MS, KI
20	*δ*-Cadinene	36.12	1513	1514	0.39 ± 0.03	2.19 ± 0.05	1.77 ± 0.04	0.73 ± 0.01	1.40 ± 0.07	---	MS, KI
21	*trans*-Calamenene	36.47	1520	1519	0.34 ± 0.02	1.53 ± 0.04	0.70 ± 0.03	---	1.01 ± 0.03	---	MS, KI
22	Junipene	41.58	1553	1555	2.48 ± 0.05	3.50 ± 0.08	2.92 ± 0.07	1.93 ± 0.03	4.58 ± 0.09	4.61 ± 0.08	MS, KI
23	*α*-Calacorene	42.05	1565	1566	---	---	---	---	0.23 ± 0.03	---	MS, KI
**Oxygenated sesquiterpenes**											
24	*cis*-Lanceol	37.23	1527	1525	---	---	0.23 ± 0.01	0.82 ± 0.03	0.66 ± 0.01	0.89 ± 0.03	MS, KI
25	Ledol	37.50	1564	1565	1.23 ± 0.03	2.40 ± 0.04	2.02 ± 0.04	0.34 ± 0.01	1.30 ± 0.03	0.64 ± 0.02	MS, KI
26	*trans*-Nerolidol	37.88	1566	1564	0.53 ± 0.02	9.03 ± 0.07	8.27 ± 0.10	5.81 ± 0.11	5.39 ± 0.21	10.14 ± 0.13	MS, KI
27	Epiglobulol	38.30	1579	1580	2.31 ± 0.06	1.58 ± 0.05	1.60 ± 0.05	2.47 ± 0.07	---	3.73 ± 0.07	MS, KI
28	Spathulenol	38.66	1580	1579	---	---	0.33 ± 0.02	---	---	0.26 ± 0.01	MS, KI
29	Caryophyllene oxide	38.79	1582	1583	0.32 ± 0.03	---	0.38 ± 0.02	0.96 ± 0.01	---	0.66 ± 0.03	MS, KI
30	Globulol	39.32	1586	1585	14.13 ± 0.09	10.57 ± 0.07	6.17 ± 0.09	18.47 ± 0.12	10.75 ± 0.08	26.42 ± 0.36	MS, KI
31	Viridiflorol	39.74	1590	1591	1.00 ± 0.03	0.48 ± 0.02	0.77 ± 0.01	1.68 ± 0.09	---	1.46 ± 0.03	MS, KI
32	Alloaromadendrene oxide-(1)	39.96	1625	1625	0.73 ± 0.03	0.55 ± 0.01	---	---	---	---	MS, KI
33	*γ*-Eudesmol	40.21	1628	1626	---	---	---	0.49 ± 0.01	1.07 ± 0.01	0.42 ± 0.02	MS, KI
34	*tau*-Muurolol	40.45	1644	1642	1.09 ± 0.05	1.72 ± 0.03	0.96 ± 0.03	0.69 ± 0.03	1.69 ± 0.03	---	MS, KI
35	Cubenol	40.98	1645	1644	2.56 ± 0.06	3.96 ± 0.06	1.99 ± 0.05	2.31 ± 0.05	2.98 ± 0.06	2.26 ± 0.04	MS, KI
36	*δ*-Cadinol	41.87	1646	1647	0.34 ± 0.03	0.73 ± 0.02	1.99 ± 0.03	1.98 ± 0.06	0.80 ± 0.03	1.65 ± 0.06	MS, KI
37	*α*-Acorenol	42.01	1655	1656	0.12 ± 0.01	0.47 ± 0.01	0.17 ± 0.01	1.13 ± 0.08	1.47 ± 0.02	1.67 ± 0.03	MS, KI
38	Eudesm-7(11)-en-4-ol	42.22	1689	1688	---	---	---	---	---	10.59 ± 0.21	MS, KI
	**Total**				99.99	99.99	99.96	99.98	99.28	99.79	

* The values represent means of the concentration (%) ± SD. RM: Red Malaysian, EQ: El-Qanater, WI: White Indian, E: Early, ESEG: El-Sabahya El-Gedida, RI: Red Indian. Rt: Retention time; KI_Lit_: Kovats retention index on a DB-5 column in reference to n-alkanes; KI_Exp_: Experimental Kovats retention index. The identification of EO components was established on the mass spectral data (MS) and Kovats indices (RI) with those of Wiley spectral library collection and NIST library databases.

## Data Availability

Not available.

## Sample Availability

Samples of the compounds are not available from the authors.
